# Effects of TLR3 and TLR9 Signaling Pathway on Brain Protection in Rats Undergoing Sevoflurane Pretreatment during Cardiopulmonary Bypass

**DOI:** 10.1155/2017/4286738

**Published:** 2017-12-27

**Authors:** Zhou Nan, Zhou Jin, Cao Huijuan, Zhang Tiezheng, Chen Keyan

**Affiliations:** ^1^Department of Anesthesiology, General Hospital of Shenyang Military Area Command, No. 83 Wenhua Road, Shenyang, Liaoning 110016, China; ^2^Department of Laboratory Animal Science, China Medical University, No. 77 Puhe Road, Shenyang North New Area, Shenyang, Liaoning 110122, China

## Abstract

**Objective:**

To investigate the effects of TLR3 and TLR9 signaling pathway on brain injury during CPB in rats pretreated with sevoflurane and its possible molecular mechanism.

**Methods:**

SD rats were randomly assigned to sham group, CPB group, and Sev group. Brain tissue was obtained at before CPB (*T*_0_), at CPB for 30 minutes (*T*_1_), 1 hour after CPB (*T*_3_), and 3 hours after CPB (*T*_5_). ELISA was used to measure S100-*β* and IL-6. Western blot was utilized to determine TLR3 and TLR9 expression. TUNEL was applied to detect neuronal apoptosis.

**Results:**

Compared with CPB group, at *T*_1_, at termination after 1 hour of CPB (*T*_2_), *T*_3_, 2 hours after CPB (*T*_4_) and *T*_5_, S100-*β* and IL-6 decreased in Sev group. Compared with CPB group, IFN-*β* were increased in Sev group, except *T*_0_. Compared with CPB group, TLR3 expression increased, and TLR9 and NF-*κ*B decreased in Sev group. The apoptotic neurons were less in Sev group than in CPB group (*P* < 0.05).

**Conclusion:**

Sevoflurane intervention can activate TLR3 and TLR9 signaling pathway, upregulate TLR3 expression and downstream TRIF expression, decrease TLR9 expression, and downregulate downstream NF-*κ*B expression in CPB rat models, thereby mitigating brain injury induced by inflammatory response during CPB.

## 1. Introduction

The emergence, application, and popularization of cardiopulmonary bypass (CPB) have led to the switch of cardiovascular surgery and significantly improve the survival rate of patients [1]. The disadvantage associated with CPB is its damage to other systems in the body. Researchers have developed various measures to reduce CPB damage to the body and significantly improve patient survival and reduce the incidence of other systemic complications, but the incidence of complications of the nervous system is relatively constant; for example, the incidences of stroke and encephalopathy are approximately 2%–5% and 10%–30%, respectively [2]. After CPB, brain injury prolongs the length of hospital stay, increases the risk of complications, consumes medical resources, and hinders the development of cardiovascular surgery.

The mechanism of brain injury after CPB is very complex; the main causes are cerebral emboli (gas, liquid, or solid), cerebral ischemic injury (such as vascular embolism, hypoperfusion, and hypoxia), and inflammatory response [3–5]. During CPB, surgical trauma stimulation, blood contact with foreign body, body endotoxin, and low temperature can activate noninfectious systemic inflammatory response syndrome [6, 7]. Thus, a large number of inflammatory cytokines enter the brain and produce brain damage. Current study of brain injury after cardiac surgery showed that a series of inflammatory factors activated and released after inflammatory response are strongly associated with brain damage after CPB.

The focus of research on reducing brain injury after CPB has shifted from trying to manage extracorporeal circulation to using a preventive strategy. These preventive strategies include the use of drug pretreatment and appropriate measures such as avoiding aortic operation, exhausting the gas in the heart cavity, and preventing air from entering the pump [8]. Pretreatment of narcotic drugs is presented on the basis of simulated ischemic preconditioning and has the same effect as ischemic preconditioning. Sevoflurane has some advantages, including rapid anesthetic induction, rapid consciousness, and low blood gas distribution coefficient and has become the current commonly used inhalation anesthetics [9]. A previous study confirmed that sevoflurane pretreatment could provide neuroprotection extensively, enhance tolerance of brain tissue to ischemic injury, and improve nerve repair after injury; thus, the neuroprotective effect of sevoflurane pretreatment has attracted much attention [10]. Present studies have shown that the protective effect of sevoflurane pretreatment on the brain may be strongly associated with the inhibition of systemic inflammatory response [11]. Bedirli et al. [12] considered that sevoflurane could improve inflammation, brain lipid peroxidation, and histological damage by downregulating TNF-*α* and IL-1*β*. Ye et al. [13] pointed out that the expression of nuclear factor-kappa B (NF-*κ*B) increased when cerebral ischemic injury occurred. In contrast, one of the possible mechanisms of sevoflurane pretreatment in brain protection is to inhibit the expression of NF-*κ*B protein. The existing studies suggest that sevoflurane is associated with inflammatory responses, but its specific molecular mechanisms are unclear.

Toll-like receptors (TLRs) recognize pathogens by pathogen-associated molecular patterns (PAMPs) in the early stage of pathogen invasion and then participate in the inflammatory response. So far, 10 human TLRs (TLR1-10) and 12 rat TLRs (TLR1-9, TLR11-13) have been identified [14]. Recent studies have found that signal transduction pathway mediated by TLR3 and TLR9, which are important members of TLRs family, may have an inseparable relationship with ischemic injury [15, 16]. He et al. [17] observed periventricular axonal injury, ependymal rupture, and activation of glial cells around the hippocampus through intracerebroventricular injection of TLR9 specific ligand nonmethylated CpG oligodeoxynucleotides in rats, indicating that TLR9 signaling pathway may induce inflammatory response in the nervous system and further cause brain damage [18]. TLR3 is an unique member of the TLR family. First, TLR3 is highly expressed in astrocytes; second, there are two immune pathways in downstream in the TLR family [10]. The vast majority of TLRs (TLR2, 4, 8, and 9) is transmitted via the MyD88 pathway [17, 19, 20]; only TLR3 depends on the TRIF pathway [21]. Pan et al. [22] verified that TLR3 preexcitation could increase the tolerance of brain tissue to ischemic injury, reduce inflammatory response, promote the production of anti-inflammatory cytokines and neuroprotective mediators, and attenuate ischemic brain injury.

Taking whether sevoflurane suppressed inflammatory lesions during CPB through activating TLR3 and TLR9 signaling pathway and protected the brain against injury as the starting point, the present study observed the effects of 2.4% sevoflurane pretreatment on brain injury and TLR3 and TLR9 signaling pathway during CPB in rat models. We also analyzed whether sevoflurane pretreatment exerted protective effect on the brain by activating TLR3 and TLR9 signaling pathway and investigated the possible molecular mechanisms so as to establish a certain basis for investigating the exact mechanism of TLR3 and TLR9 signaling pathway in the neuroprotection of sevoflurane treatment in rats undergoing CPB.

## 2. Materials and Methods

### 2.1. Experimental Animals and Groups

Sixty-four adult male Sprague-Dawley rats weighing 350–450 g were provided by the Experimental Animal Center of the General Hospital of Shenyang Military of China (approval number: 20120002). The protocols were approved by the Animal Experiment Ethics Committee of the General Hospital of Shenyang Military. The rats were fasted for 12 hours before surgery. All rats were randomly assigned to sham surgery group (sham, *n* = 8), CPB group (CPB, *n* = 24), and sevoflurane pretreatment group (Sev, *n* = 32).

### 2.2. Model Establishment and Interventions

#### 2.2.1. Preparation of a Rat Model of CPB Primed without Blood

Rat model of CPB was performed as previously reported [23]. Rats were intraperitoneally anesthetized with 10% chloral hydrate 300 mg/kg. Direct orotracheal intubation was conducted using a 16 G venous catheter. Mechanical ventilation was carried out with a small animal ventilator at a frequency of 60 times/min, oxygen flow of 11/min, tidal volume of 3 ml/kg, and inspiratory-to-expiratory ratio of 1 : 1.5. Rat heart rate, saturation of blood oxygen, and rectal temperature were monitored using a monitor.

The hair was shaved at the site of puncture. After disinfecting and cutting, blood vessels were isolated and exposed. 23 G venous catheter on the left side was connected to the microinfusion pump. 24 G femoral artery catheter on the left side was connected to the monitor to real-time detect blood pressure. A self-made multi-empty drainage needle (16 G) was inserted in the right internal jugular vein and reached the right atrium as blood pressure drainage during CPB. Right femoral artery puncture catheter was fixed for perfusion during CPB. The sites of puncture were connected with a drainage tube, a self-made blood reservoir, a constant flow peristaltic pump, a silica gel pipeline, and a rat membrane oxygenator to establish CPB circuit. The left femoral vein was chosen as the site of systemic heparinization in rats and injected with heparin sodium injection 300 IU/kg. When ACT reached 400–500s, it was considered up to standard. After the rats were primed without blood, rat models of CPB on a beating heart were established. Priming solution consists of 6% hydroxyethyl starch 12 ml, 5% sodium bicarbonate 2 ml, 20% mannitol 1 ml, and heparin sodium 150 IU/kg.

Membrane oxygenator was immediately used after CPB. The bypass speed was 35 ml/(kg·min) (low flow) at beginning and gradually increased to 100–120 ml/(kg·min) (full flow). In order to prevent the formation of air embolism, 1-2 ml blood was kept in the blood container. MAP was kept higher than 60 mmHg; PH was at 7.35–7.45 level; PaCO_2_ was between 35 and 45 mmHg; Hct was greater than 0.25. Vasoactive drugs and fluid supplement were given during the operation to maintain the circulatory stability in rats. At 1 hour after CPB, extracorporeal circulation was gradually terminated, and mechanical ventilation was carried out. At 2 hours after termination of the circulation, stable vital signs of rats indicated successful model establishment.

#### 2.2.2. Treatments in Each Group

In the sham group, tracheal intubation and mechanical ventilation were only performed in the right femoral artery. Bypass was not conducted in the right internal jugular vein puncture catheterization. In the CPB group, models of CPB were established. In the Sev group, after pretreatment with 2.4% sevoflurane for 1 hour, CPB models were established. In the CPB and Sev groups, CPB was performed for 1 hour.

Sevoflurane pretreatment was as follows: Soda lime was paved under the gauze at the bottom of the pretreatment box. Two holes were provided at both ends of the box. One end connected to anesthesia ventilator, and one end connected to the gas collection port of the monitor to real-time monitor oxygen and sevoflurane concentrations. The temperature in the box was maintained at 35–37°C. After the rats were placed in the box, oxygen and sevoflurane flow meter was opened to adjust their concentrations. When sevoflurane was kept at the needed concentrations, pretreatment time was counted for 1 hour.

#### 2.2.3. Specimen Collection and Processing

Arterial and venous blood was collected before CPB (*T*_0_), at CPB for 30 minutes (*T*_1_), termination after 1 hour of CPB (*T*_2_), 1 hour after CPB (*T*_3_), 2 hours after CPB (*T*_4_), and 3 hours after CPB (*T*_5_). Eight rats from each group were sacrificed at *T*_0_, *T*_1_, *T*_3_, and *T*_5_ in the Sev and CPB groups, and brain tissue was obtained.

#### 2.2.4. Brain Tissue

After the heart was exposed, the circulatory system was perfused and washed with 250–400 ml of physiological saline. The skull was broken off and the brain was obtained on the ice plate. The brain was cut into two pieces through the median sagittal line. The hippocampi were isolated and stored in –80°C (left side) and 10% neutral formalin (right side).

#### 2.2.5. Venous Blood Serum and Arterial Blood Gas

Venous blood was centrifuged at 3000 rpm for 10 minutes, and serum was collected and stored at –80°C. Blood was collected from left femoral artery and subjected to blood gas analysis, 1.3 ml/time. After sampling, an equal volume of 6% hydroxyethyl starch was intravenously injected.

#### 2.2.6. Cell Apoptosis as Measured by Terminal Deoxynucleotidyl Transferase dUTP Nick End Labeling (TUNEL) Assay In Situ

Paraffin sections were dewaxed and hydrated, treated with proteinase K for 15 minutes, washed four times with water at room temperature, treated with H_2_O_2_-PBS mixture for 15 minutes, and washed three times with PBS, each for 5 minutes. These sections were then treated with TUNEL for 10 minutes, stop buffer for 30 minutes, washed with PBS, incubated with horseradish peroxidase for 30 minutes, and washed with PBS. Afterwards, sections were incubated with DAB-H_2_O_2_ mixture for 3 minutes, washed with PBS, counterstained with methyl green for 10 minutes, and washed three times with distilled water and three times with N-butanol, each for 1 minute. Subsequently, the sections were dehydrated with xylene, dried, and mounted. Nuclei of normal neurons were stained blue using hematoxylin, considering as negative cells. Nuclei of injured neurons were stained brown, with the presence of DNA breakage, considering positive cells. Six sections were selected from each group and five fields of each section were selected at 400x magnification. Mean integrated optical density values of injured neurons were analyzed using Metamorph Dplo BX41 image analysis system.

#### 2.2.7. Serum S100-*β*, IFN-*β*, IL-6, TLR3, and TRIF Concentrations in Rats as Detected by Enzyme Linked Immunosorbent Assay (ELISA)

Sample (50 *μ*l) was added in each well after standard substance was diluted. The sample (50 *μ*l) diluted five times was added and incubated at 37°C for 30 minutes. The washing liquid was added to the waste liquid hole for 30 seconds, repeatedly for five times. ELISA reagents (50 *μ*l) were added in each well, and developers A and B (each 50 *μ*l) were added and incubated at 37°C for 15 minutes. Stop buffer (50 *μ*l) was added for 15 minutes. Absorbance values were measured at 450 nm, and results were analyzed.

#### 2.2.8. TLR3 and TLR9 Expression as Measured by Western Blot Assay

The hippocampus was lysed with 1 ml/100 mg lysate and centrifuged at 12000 rpm and 4°C for 15 minutes. The supernatant was incubated with loading buffer, subjected to electrophoresis, and transferred onto the membrane for 90 minutes. The membrane was blocked with 5% defatted milk powder for 2 hours and incubated with approximately 1 ml of TLR3 antibody or TLR9 antibody (Abcam, USA) at room temperature for 2 hours. After removal of primary antibody, the membrane was washed four times with phosphate-buffered saline/Tween. The incubation with secondary antibody was the same as before. After visualization, results were analyzed using Quantity One software.

#### 2.2.9. Statistical Analysis

Data were analyzed with SPSS 19.0 software. Measurement data were expressed as the mean ± standard deviation. Intergroup comparison was completed using one-way analysis of variance. Intragroup comparison was finished using repeated measures analysis of variance. A value of *P* < 0.05 was considered statistically significant.

## 3. Results

### 3.1. Life Indicators and Blood Gas Analysis in Rats of Each Group

MAP, HR, rectal temperature, pH value, PaCO_2_, PaO_2_, BE value, and K^+^ were not significantly different among groups at various time points (*P* > 0.05). MAP decreased to 74 mmHg during CPB (versus *T*_0_; *P* < 0.05) and gradually increased to 99 mmHg after CPB (versus *T*_0_; *P* > 0.05). HR was 296.51 bpm/min during CPB (versus *T*_0_; *P* < 0.05) and gradually recovered to 309.20 bpm/min after CPB (versus *T*_0_; *P* > 0.05). Hct decreased to 27.49 immediately during CPB (*P* < 0.05). pH value, PaCO_2_, PaO_2_, BE value, and K^+^ levels were relatively stable at various time points and were not significantly different as compared with *T*_0_ (*P* > 0.05; Table 1).

### 3.2. Serum S100-*β* Concentrations in Rats of Each Group

**Figure 1:**
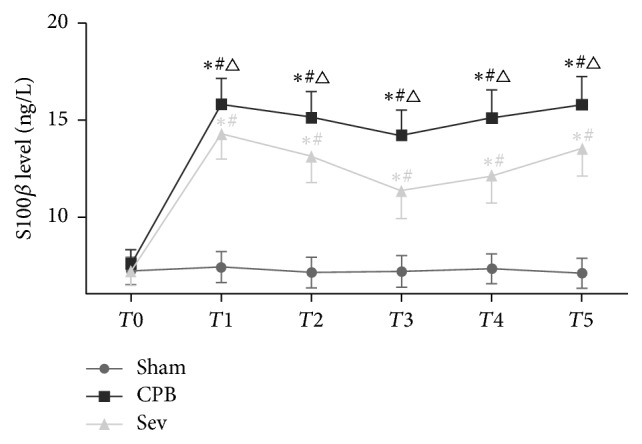
After establishment of the CPB model, sevoflurane treatment and ELISA were used to detect the serum S100 - *β* level in each group rats at different times. Data collected from sham group, CPB group, and Sev group. Compared with *T*0, ^#^*P* < 0.05; compared with sham group ^*∗*^*P* < 0.05; compared with CPB group, ^△^*P* < 0.05.

Serum S100-*β* concentrations significantly increased during CPB and gradually diminished after CPB in the CPB group (versus *T*_0_; *P* < 0.05). Compared with the sham group, serum S100-*β* concentrations significantly increased at *T*1–*T*5 in the CPB and Sev groups (*P* < 0.05). Compared with the CPB group, serum S100-*β* concentrations significantly reduced at *T*_1_–*T*_5_ in the Sev group (*P* < 0.05, Figure 1).

### 3.3. ELISA Results of IL-6 Concentrations in Rats of Each Group

**Figure 2:**
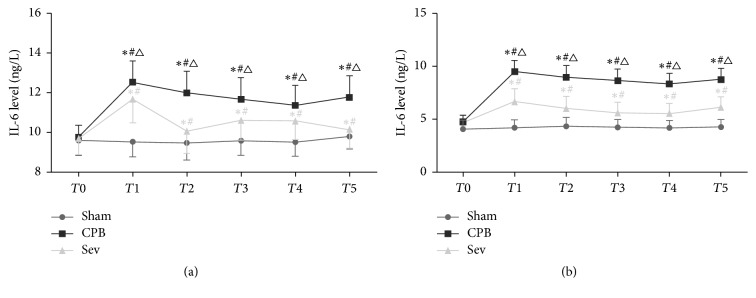
ELISA used to detect the IL-6 level in each group rats at different times. Data collected from sham group, CPB group, and Sev group. (a) Serum IL-6 level. (b) Brain IL-6 level. Compared with *T*0, ^#^*P* < 0.05; compared with sham group ^*∗*^*P* < 0.05; compared with CPB group, ^△^*P* < 0.05.

Compared with the sham group, serum (Figure 2(a)) and brain (Figure 2(b)) IL-6 concentrations significantly increased in the CPB and Sev groups at *T*_1_–*T*_5_ (versus T_0_; *P* < 0.05, *P* < 0.05). Compared with the CPB group, serum and brain IL-6 concentrations significantly diminished in the Sev group at *T*_1_–*T*_5_ (*P* < 0.05).

### 3.4. ELISA Results of IFN-*β* Concentrations in Rats of Each Group

**Figure 3:**
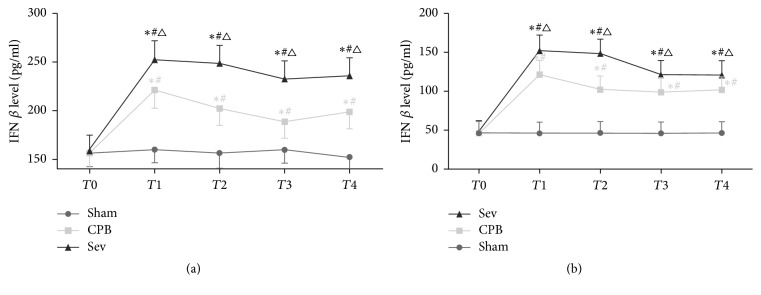
ELISA used to detect the IFN-*β* level in each group rats at different times. Data collected from sham group, CPB group, and Sev group. (a) Serum IFN-*β* level. (b) Brain IFN-*β* level. Compared with *T*0, ^#^*P* < 0.05; compared with sham group ^*∗*^*P* < 0.05; compared with CPB group, ^△^*P* < 0.05.

Compared with the sham group, IFN-*β* concentrations increased at *T*1–*T*5, significantly increased at *T*1, and gradually reduced at *T*2 in the CPB and Sev groups (*P* < 0.05). Compared with the CPB group, besides *T*0, serum and brain IFN-*β* concentrations significantly increased in the Sev group at various time points (*P* < 0.05; Figures 3(a) and 3(b)).

### 3.5. ELISA Results of TLR3 Protein Expression in the Rat Hippocampus of Each Group

**Figure 4:**
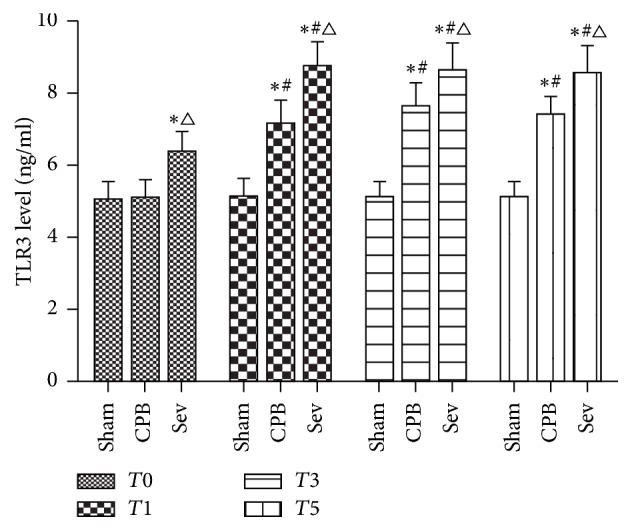
The expression of TLR3 protein in hippocampus of rats by ELISA. Data collected from sham group, CPB group, and Sev group. Compared with *T*0, ^#^*P* < 0.05; compared with sham group ^*∗*^*P* < 0.05; compared with CPB group, ^△^*P* < 0.05.

Compared with the sham surgery group, TLR3 protein expression increased at *T*_1_, *T*_3_, and *T*_5_, significantly increased at *T*_1_, and gradually diminished at *T*_3_ in the CPB and sevoflurane pretreatment groups (versus *T*_0_; *P* < 0.05, *P* < 0.05). Compared with the CPB group, TLR3 expression significantly increased at *T*_1_, *T*_3_, and *T*_5_ in the sevoflurane pretreatment group (*P* < 0.05; Figure 4).

### 3.6. ELISA Results of TRIF Protein Expression in the Rat Hippocampus of Each Group

**Figure 5:**
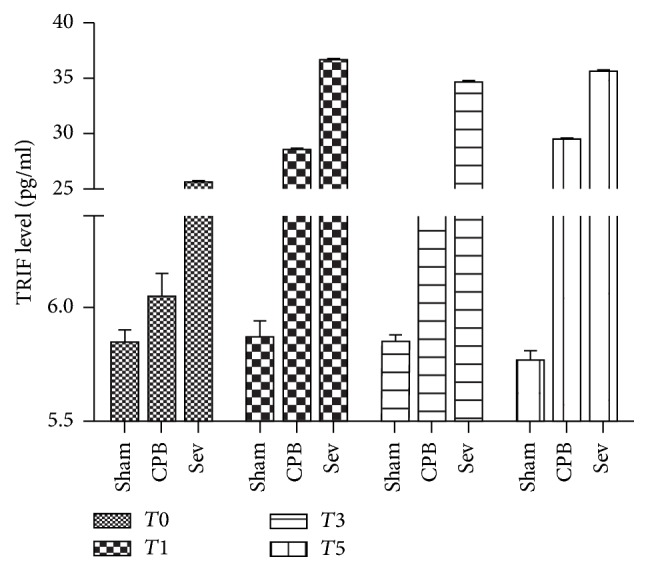
The expression of TRIF protein in hippocampus of rats by ELISA. Data collected from sham group, CPB group, and Sev group.

Compared with the sham surgery group, TRIF protein expression increased at *T*_1_, *T*_3_, and *T*_5_, significantly increased at *T*_1_, and diminished at *T*_3_ in the CPB and sevoflurane pretreatment groups (versus *T*_0_; *P* < 0.05). Compared with the CPB group, TRIF protein expression significantly increased at *T*_1_, *T*_3_, and *T*_5_ in the sevoflurane pretreatment group (*P* < 0.05; Figure 5).

### 3.7. Western Blot Assay Results of TLR3, TRIF, TLR9, and NF-*κ*B Protein Expression in the Rat Hippocampus of Each Group

**Figure 6:**
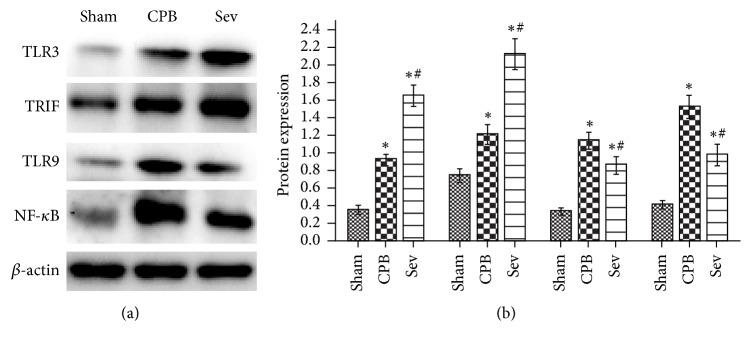
Western blot used to detect the TLR3 and TLR9 signaling pathway related proteins TLR3, TRIF, and TLR9, NF-*κ*B expression in hippocampus of rats. Data collected from sham group, CPB group, and Sev group. Compared with sham group ^*∗*^*P* < 0.05; compared with CPB group, ^#^*P* < 0.05.

Compared with the sham group, TLR3, TRIF, TLR9, and NF-*κ*B expression significantly increased in the CPB and Sev groups (*P* < 0.05). Compared with the CPB group, TLR3 and TRIF expression significantly increased and TLR9 and NF-*κ*B expression significantly decreased in Sev group (*P* < 0.05; Figure 6).

### 3.8. Neuronal Apoptosis in the Hippocampus as Measured by TUNEL Assay

**Figure 7:**
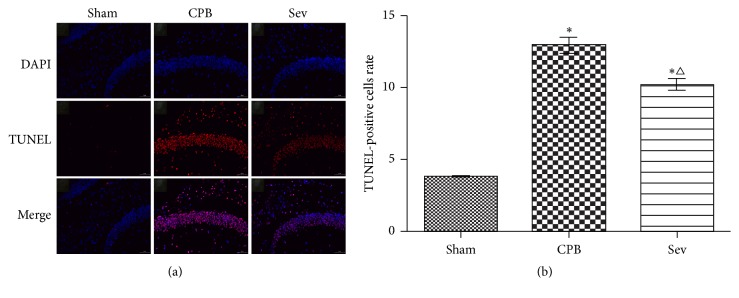
TUNEL was used to detect the apoptotic positive cells (400x). Data collected from sham group, CPB group, and Sev group. Compared with sham group ^*∗*^*P* < 0.05; compared with CPB group, ^△^*P* < 0.05.

The number of positive cells was less in the sham group. Compared with the sham group, the number of positive cells was significantly increased in the CPB and Sev groups (*P* < 0.05). The number of positive cells significantly decreased in the Sev and CPB groups (*P* < 0.05; Figure 7).

## 4. Discussion

This study established rat models of CPB primed without blood after 1-hour pretreatment with 2.4% sevoflurane. Serum S100-*β* protein was selected as biochemical marker for brain injury. 1.0 MAC sevoflurane has been extensively used in the clinic. Previous studies have shown that 2.4% sevoflurane was equivalent to rat 1.0 MAC sevoflurane [24, 25]. Hu et al. [26] demonstrated that sevoflurane could mitigate cerebral ischemia/reperfusion injury after 1-hour pretreatment with 2.4% sevoflurane. Sevoflurane is commonly used in the rat models of middle cerebral artery but seldom used in rat models of CPB. In accordance with the results of our preliminary experiments, the concentration of sevoflurane was 2.4% in this study.

S100-*β* is a sensitive neuron-specific protein, and its expression is very low in the normal brain. Only when brain tissue is damaged is S100-*β* activated early and expressed rapidly [27]. Because the permeability of cell membrane and blood-brain barrier increased, S100-*β* can be released into the cerebrospinal fluid and the systemic circulation through the blood-brain barrier; thus, the detection of S100-*β* concentrations in peripheral blood can sensitively reflect brain injury [28]. The researchers therefore regard S100-*β* protein as a marker that reflects brain injury. Similarly, in the study of CPB, S100-*β* is considered as a specific marker for early brain injury in CPB [29, 30]. The hippocampus is more sensitive to cerebral ischemia and hypoxia compared with other parts of the brain, and ischemia and hypoxia are more likely to cause neuronal damage, so the hippocampus is selected in this study [31].

In this study, serum S100-*β* levels were close to normal value before CPB and were low in each group. After CPB, S100-*β* levels were still low in the sham group, indicating that simple intubation did not cause significant brain injury. Compared with the sham group, serum S100-*β* levels obviously increased during CPB in the CPB and Sev groups, and S100-*β* remained in a remarkably high level after CPB. Singh et al. [32] found that, under intravenous anesthesia of sevoflurane and isoflurane, S100-*β* protein concentrations maximally increased after CPB in patients undergoing coronary artery bypass grafting, and S100-*β* concentrations were minimal in the Sev group, which was consistent with our results. These findings suggest that CPB-induced brain injury in rats through some mechanisms.

During CPB, various factors, such as nonphysiological perfusion, local oxygen supply, and insufficient blood supply, can lead to the release of large numbers of proinflammatory cytokines from lymphocytes, resulting in systemic inflammatory response syndrome [33], which can cause great damage to the brain. Therefore, inflammatory lesion is an important cause of cognitive impairment after CPB. How to effectively reduce inflammatory lesion during CPB is the foothold of our research.

Ramlawi et al. [34] demonstrated that serum IL-1 levels remarkably increased, and the changes in concentration were positively associated with cognitive decline in patients with postoperative cognitive decline in the early stage after coronary artery bypass grafting and valve replacement. Ashraf et al. [35] thought that, during CPB, S100-*β* protein expression was positively associated with IL-6 expression; IL-6 expression had promoting effect on S100-*β* protein expression; inflammatory mediators participate in and aggravate brain injury. Vila et al. [36] confirmed that IL-6 was strongly associated with area of acute cerebral infarction; serum IL-6 concentration and cerebral infarction area showed the same trend and were independent risk factors for cerebral infarction. In this study, IL-6 concentrations obviously increased in the CPB group compared with the sham group from the beginning of CPB and then gradually reduced after CPB but were still high, indicating that CPB definitely started the inflammatory response, which was consistent with the results from previous studies. Compared with CPB and sham groups, IL-6 concentrations increased during CPB in the Sev group; however, IL-6 concentrations were still lower in the Sev group than in the CPB group at various time points. After CPB, IL-6 concentrations were remarkably decreased, and the descent speed was faster than that in the CPB group. Furthermore, the trend of IL-6 concentration was the same as that of S100-*β*. S100-*β* concentrations were lower in the Sev group than in the CPB group, suggesting that sevoflurane mitigated brain injury induced by inflammatory response during CPB through inhibiting IL-6 expression. Bedirli et al. [12] believed that sevoflurane pretreatment could diminish the concentrations of inflammatory factors (TNF-*α* and IL-1*β*) during ischemia and reperfusion, lessen inflammatory response-induced brain injury, and exert protective effect on the brain, which was consistent with our results.

The establishment of CPB causes the brain tissue to undergo multiple noxious stimuli, such as hypoxia and ischemia of histiocytes induced by abnormal perfusion and subsequent reperfusion injury, activation of systemic complement system by foreign bodies such as pipes, and extremely released endotoxin [37]. These noxious stimuli can strongly induce the formation of pathophysiological processes characterized by inflammatory response through a variety of factors and pathways [38]. TLR9 signaling pathway, as an important hinge between innate immunity and acquired immunity, can play an immune regulatory role in the early stage of pathogen invasion [39]. In ischemic brain injury, TLR9 activates the downstream signaling molecule NF-*κ*B by binding to specific ligands and participates in inflammatory response [40]. Trop et al. [41] found that CPB increased inflammatory response, induced high expression of interferon-gamma (INF-*γ*), TNF-*α*, IL-6, IL-8, and IL-10, activated TLRs signaling pathway, and upregulated IRAK-4 and NF-*κ*B expression. Mahle et al. [42] performed a study on the relationship between inflammatory response and clinical prognosis in neonates after CPB and found that neonatal cardiac surgery can cause complex and extensive inflammatory response, leading to the increase of INF-*γ*, TNF-*α*, IL-2, IL-5, IL-6, IL-8, IL-10, and IL-13. In the present study, the establishment of CPB activated inflammatory TLR9 signaling pathway in the brain and increased NF-*κ*B expression. This indicates that TLR9 signaling pathway is involved in the pathological process of cerebral ischemic injury, which was consistent with Trop et al.'s study [41]. This result may be because CPB can cause hypoxia and ischemia in brain tissue; blood-brain barrier is damaged; apoptosis and necrosis occur in neuron cells; the damaged tissues and necrotic cells can release some molecules as endogenous activators of TLR9 to activate the TLR9 signaling pathway. For example, cleavage of DNA fragments can act as ligands on TLR9 receptors and activate the TLR9 signaling pathway [43]. Taken together, inflammatory response induced by CPB may be associated with the signaling pathway of TLR9 inflammatory response.

TLR3 is the only complete, single TLR through the TRIF-dependent pathway. TLR3 is expressed throughout the central nervous system and prominently highly expressed in astrocytes. Astrocytes account for the highest proportion of cells in the central nervous system, so TLR3 is particularly sensitive to injury [44]. Relative to the response of TLR to brain injury caused by inflammatory response during ischemia, if certain stimuli are given in advance to activate TLR, it can provide a powerful neuroprotective effect by reducing inflammatory response [45]. Previous studies have confirmed that pretreatment with lipopolysaccharide or upregulation of TRIF expression by ischemic preconditioning can ultimately increase the release of anti-inflammatory cytokines, inhibit the release of nuclear factor-kappa B- (NF-*κ*B-) mediated proinflammatory cytokines, thus playing a protective effect on the brain [8, 9]. Nhu et al. [46] found that lipopolysaccharide pretreatment could upregulate TLR3 expression, thereby increasing the expression in the downstream TRIF pathway. Because TLR3 signaling is completely through the TRIF pathway, activation of TLR3 expression may contribute to the balance of proinflammatory and anti-inflammatory cytokines, resulting in neuroprotection. Pan et al. [47] demonstrated that the use of TLR3 agonist polyinosinic:polycytidylic acid could stimulate TLR3 expression, inhibit the release of proinflammatory cytokines NF-*κ*B and IL-6, and alleviate the damage to nerve cells.

In the current study, the trend of TRIF expression is basically the same as that of TLR3, and TLR3 expression is confirmed by the downstream pathway. Because TLR3 signaling is completely through the TRIF pathway, the activation of TLR3 expression may help to improve the balance of proinflammatory and anti-inflammatory cytokines that are broken by CPB, to search for novel balances and to produce neuroprotective effects. IL-6 concentrations and S100-*β* protein concentrations showed the same trend, suggesting that IL-6 concentrations increased during CPB, accompanied by or aggravating the occurrence and development of brain injury. Before CPB, IL-6 concentrations were lower in the Sev group than in the sham group. The trend of IL-6 change in the Sev group was approximately identical to that of CPB group, but IL-6 concentrations were obviously lower. In this study, after pretreatment with sevoflurane in rat models of CPB, sevoflurane pretreatment activated TLR3 expression, increased TLR3 and TRIF levels, and inhibited the production of S100*β* and IL-6. Sevoflurane pretreatment is similar to ischemic preconditioning, can activate TLR3 and TRIF protein expression, enhance the tolerance of brain tissue to inflammatory lesion caused by CPB, lessen IL-6 release, and reduce inflammatory factors-induced damage. Results from the present study confirmed that TLR3 participated in the brain protection of sevoflurane pretreatment and provided a new target for the prevention and improvement of CPB-induced brain injury in clinic.

In conclusion, sevoflurane pretreatment has protective effect against brain injury after CPB. Sevoflurane pretreatment can activate TLR3 and TLR9 signaling pathway, upregulate TLR3 expression and TRIF expression, decrease TLR9 expression, and downregulate NF-*κ*B expression and inhibited the production of S100*β* and IL-6, thereby lessening CPB inflammation-induced brain injury.

## Figures and Tables

**Table 1:** MAP, HR, rectal temperature, and blood gas analysis results in rats of each group $(\overset{-}{x}\pm s)$.

Item	*T*_0_	*T*_1_	*T*_2_	*T*_3_	*T*_4_	*T*_5_
MAP (mmHg)	103.80 ± 15.91	74.11 ± 7.06^*∗*^	73.06 ± 5.36^*∗*^	90.60 ± 6.85	98.97 ± 7.04	99.17 ± 7.16
HR (/min)	312.69 ± 12.13	296.51 ± 11.67^*∗*^	293.60 ± 8.83^*∗*^	295.51 ± 7.49	307.83 ± 8.04	309.20 ± 7.65
*T* (°C)	36.96 ± 0.33	34.42 ± 0.26^*∗*^	35.22 ± 0.33^*∗*^	36.61 ± 0.32	36.85 ± 0.27	36.87 ± 0.41
pH	7.40 ± 0.03	7.37 ± 0.02	7.37 ± 0.02	7.38 ± 0.02	7.39 ± 0.03	7.39 ± 0.03
PaCO_2_ (mmHg)	39.57 ± 2.82	39.46 ± 3.18	40.29 ± 3.04	38.63 ± 2.81	38.83 ± 2.86	39.71 ± 2.90
PaO_2_ (mmHg)	312.06 ± 22.65	307.71 ± 14.83	302.31 ± 21.57	304.57 ± 11.18	307.29 ± 14.43	307.69 ± 15.98
BE (mmol/L)	−1.2 ± 1.45	−2.7 ± 1.52	−2.4 ± 1.63	−2.1 ± 1.24	−1.3 ± 1.58	−2.6 ± 1.59
Hct	39.37 ± 1.97	27.94 ± 1.70^*∗*^	31.09 ± 1.76^*∗*^	33.51 ± 1.77^*∗*^	33.06 ± 1.61^*∗*^	33.23 ± 1.94^*∗*^
K^+^ (mmol/L)	4.16 ± 0.43	4.10 ± 0.37	4.27 ± 0.41	4.15 ± 0.36	4.17 ± 0.39	4.16 ± 0.33

*Note*. ^*∗*^*P* < 0.05, versus T_0_.
